# A very rare complication of ventriculoperitoneal shunt of cerebrospinal fluid

**DOI:** 10.11604/pamj.2019.34.127.9497

**Published:** 2019-11-05

**Authors:** Hilal Abboud, Abdessamad Elouahabi

**Affiliations:** 1 Department of Neurosurgery, Ibn Sina Hospital Medical School, Mohamed V University, Rabat, Maroc

**Keywords:** Hydrocephalus, shunt, complication

## Image in medicine

Hydrocephalus is defined as an active dilation of cerebral ventricles secondary to a hydro-dynamic disorder of the cerebrospinal fluid (CSF). It is a common condition in pediatric neurosurgery, for which, there are several medical and surgical treatment options. The ventriculoperitoneal shunt (VPS) is the most used method, it consists to drain excess cerebrospinal fluid from the ventricles to the peritoneal cavity using a valve. Infection is the most serious complication, it occurs in 10% of cases, other complications can be noted like hemorrhage, hyperdrainage or valve malfunction. We report a rare case of a complicated VPS three months after surgery, by a distal catheter externalization on para-spinal level in 6 months patient, who was operated for malformative hydrocephalus, and which benefited on VPS with immediate good outcome. The patient presents to the emergency three months after the establishment of the VPS, she is conscious and afebrile at admission, with a normal cranial perimeter, operative scars are clean, the valve is palpable at the right mastoid and the distal catheter under the skin along its path, the distal tip is externalized several centimeters through the skin to the right para-spinal level, the VPS is removed. Biochemical and cytobacteriological analysis of the CSF and the ventricular catheter didn’t objectify meningitis. A brain CT scan is subsequently conducted to assess the degree of hydrocephalus and decide a ventricular shunt.

**Figure 1:**
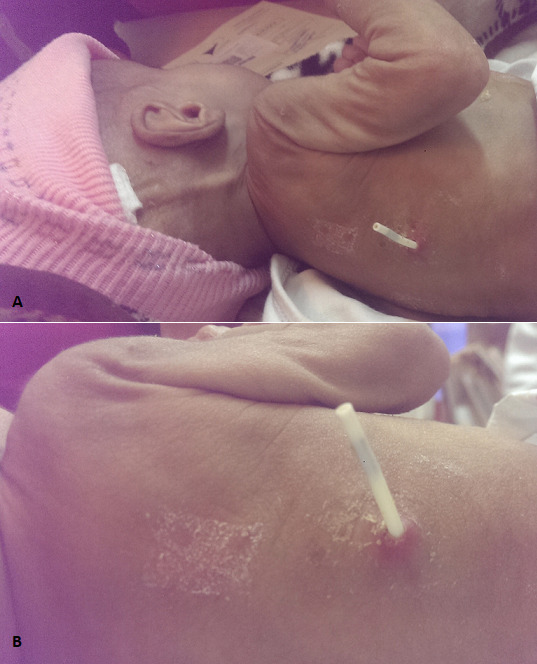
A) The distal tip of catheter externalised on paraveretebral level; B) Externalisation of several centimeters without underlying collection

